# Urinary Tract Infections due to Multidrug-Resistant Enterobacteriaceae: Prevalence and Risk Factors in a Chicago Emergency Department

**DOI:** 10.1155/2013/258517

**Published:** 2013-10-31

**Authors:** Thana Khawcharoenporn, Shawn Vasoo, Kamaljit Singh

**Affiliations:** ^1^Division of Infectious Diseases, Faculty of Medicine, Thammasat University, Pathum Thani 12120, Thailand; ^2^Section of Infectious Diseases, Rush University Medical Center, Chicago, IL 60612, USA

## Abstract

*Background*. Selection of empiric antibiotics for urinary tract infections (UTIs) has become more challenging because of the increasing rates of multidrug-resistant Enterobacteriaceae (MDRE) infections. *Methods*. This retrospective study was conducted to determine antibiotic resistance patterns, risk factors, and appropriate empiric antibiotic selection for MDRE UTIs. Adult patients seen in the Emergency Department (ED) with Enterobacteriaceae UTIs during 2008-2009 were identified from review of microbiology records. MDRE were defined as organisms resistant to at least 3 categories of antibiotics. *Results*. There were 431 eligible patients; 83 (19%) had MDRE UTIs. Resistance rates for individual antibiotics among MDRE UTIs were significantly greater than non-MDRE UTIs: levofloxacin, 72% versus 14%; TMP-SMX, 77% versus 12%; amoxicillin-clavulanate, 35% versus 4%; nitrofurantoin, 21% versus 12%, and ceftriaxone, 20% versus 0%. All Enterobacteriaceae isolates were susceptible to ertapenem (MIC ≤ 2 mg/L). Independent risk factors for MDRE UTI were prior fluoroquinolone use within 3 months (adjusted odds ratio (aOR) 3.64; *P* = 0.001), healthcare-associated risks (aOR 2.32; *P* = 0.009), and obstructive uropathy (aOR 2.22; *P* = 0.04). *Conclusion*. Our study suggests that once-daily intravenous or intramuscular ertapenem may be appropriate for outpatient treatment of ED patients with MDRE UTI.

## 1. Introduction

Enterobacteriaceae are the most common cause of urinary tract infections (UTIs) in both community and healthcare settings. Selection of empiric antibiotic therapy for UTIs is therefore often based on the institutional susceptibility profiles of the Enterobacteriaceae [1]. Recent guidelines from the Infectious Diseases Society of America recommended that empiric antibiotic therapy for UTIs should be based on local resistance data, drug availability, and antibiotic intolerance/allergy history of treated patients [1, 2]. For uncomplicated cystitis, nitrofurantoin or trimethoprim-sulfamethoxazole (TMP-SMX, if local resistance ≤ 20%) can be used empirically, while fluoroquinolones, ceftriaxone, aminoglycosides, and carbapenems are appropriate for pyelonephritis and complicated UTIs.

The increase in rates of antibiotic resistance among Enterobacteriaceae has posed challenges in choosing empiric regimens, especially when infections due to multidrug-resistant Enterobacteriaceae (MDRE) are suspected or endemic [3]. In the past decade, emerging resistance among the Enterobacteriaceae due to extended-spectrum beta-lactamases (ESBL) has been reported worldwide, including in Chicago [4]. In addition, other mechanisms of resistance among urinary pathogens including *Klebsiella pneumoniae* carbapenemases (KPC) and New Delhi metallo-beta-lactamases (NDM)-1 have also been increasingly described [5, 6]. Infections caused by these MDRE require timely and appropriate antibiotic therapy to improve patients' survival [7].

The treatment of UTIs in the Emergency Department (ED) poses a unique challenge as patients often present with a spectrum of illness severity and comorbidities. Some patients have had recurrent UTIs with multiple antibiotic exposures, failed empiric outpatient treatment, or healthcare-associated (HA) infection (including MDRE) risks [8]. As a consequence, patients are more likely to have urine cultures obtained to guide therapy; however, initial empiric antibiotic selection is frequently based on inpatient antibiograms which may overestimate antibiotic resistance. The objective of this study was to determine prevalence of patterns of antibiotic resistance, risk factors, and appropriate empiric therapy for MDRE UTIs among ED patients.

## 2. Methods

### 2.1. Study Design, Setting, and Population

This is a structured, retrospective, observational study. All patients aged ≥ 12 who were evaluated at Rush University Medical Center (RUMC) ED from 1 August 2008 to 31 March 2009 with positive urine cultures were eligible for the study. RUMC is a 613-bed tertiary academic center in Chicago with over 48,000 ED visits annually. Patients were identified by daily review of urine specimens sent to the microbiology laboratory during the study period. In RUMC ED, urine cultures are performed in the majority of patients suspected of UTI or in those with positive urinalysis for leukocyte esterase and/or nitrite. Patients who had urine cultures with significant enterobacteriaceae growth ≥10^4^ CFUs/mL were included for chart review. Only the first episode of the positive urine culture per patient was included in our analysis. The study was approved by the institutional review board of RUMC.

### 2.2. Study Protocol and Definitions

Chart review was performed exclusively using our electronic medical record system (Epic; Wisconsin, USA) by two independent reviewers (T. Khawcharoenporn and S. Vasoo). These reviewers were trained in data abstraction procedure. The collected data included ethnicity, demographics, pregnancy status, underlying medical comorbidities, relevant surgical history including urological procedure and obstructive uropathy, prior UTI, prior antibiotic use within 3 months, UTI diagnoses, empiric treatments, causative bacteria, and antibiotic susceptibility. Healthcare-associated risks were defined as presence of chronic indwelling urinary catheters, healthcare exposure including hospital stay for at least 48 hours, nursing home or long-term care facility residence, regular hemodialysis clinic visits, or undergoing urological procedures within the past 3 months [9, 10]. The diagnosis of UTI (cystitis, pyelonephritis, and urosepsis) was determined solely by the treating physician. Patients with asymptomatic bacteriuria or urinary tract colonization were excluded from the study. Identification and antibiotic susceptibilities of causative bacteria were determined using MicroScan Walkaway (Siemens, CA). The minimum inhibitory concentration (MIC) breakpoints for resistance were based on Clinical Laboratory Standards Institute criteria: levofloxacin, ≥8 mg/L; TMP-SMX, ≥4/76 mg/L; nitrofurantoin, ≥128 mg/L; gentamicin, ≥16 mg/L; amikacin, ≥64 mg/L; ampicillin, ≥32 mg/L; amoxicillin-clavulanate, ≥32/16 mg/L; piperacillin-tazobactam, ≥128/4 mg/L; ceftriaxone, ≥64 mg/L; cefepime, ≥32 mg/L, and ertapenem, ≥8 mg/L [11]. MDRE isolates were defined as isolates that were resistant or intermediate susceptible to ≥3 of the following antimicrobial categories: (1) penicillins ± beta-lactamase inhibitors (PBI); (2) cephalosporins either ceftriaxone or cefepime; (3) carbapenems; (4) fluoroquinolones; (5) gentamicin or amikacin; (6) TMP-SMX; and (7) nitrofurantoin [12, 13]. Enterobacteriaceae isolates that did not meet the criteria were considered to be non-MDRE. Results of interest included the prevalence of each Enterobacteriaceae, antibiotic resistance rates, resistance patterns of and risk factors for MDRE UTIs. 

### 2.3. Statistical Analysis

All analyses were performed using SPSS, version 15.0 (SPSS, Chicago, Illinois). Categorical variables were compared using Pearson's *χ* ^2^ or Fisher's exact test as appropriate. Continuous variables were compared using Mann Whitney *U* test. All *P* values were 2-tailed; *P* values less than 0.05 were considered statistically significant. Risk factors for MDRE UTI were identified by comparison of variables between the patients infected with MDRE and non-MDRE bacteria. Variables that were present inmore than 10% of patients at a significance level of *P* less than 0.20 or that had a prior clinical significance (e.g., recent antibiotic use and healthcare-associated risks including presence of indwelling catheters and resident of a long-term care facility) [12, 14, 15] were entered into stepwise backward logistic regression models. Significant variables that were thought to be covariates were grouped, and only one variable from each group was chosen for model entry. The model's overall robustness was confirmed by Hosmer-Lemeshow goodness-of-fit statistic. Adjusted odd ratios (aORs) and 95% confidence intervals (CIs) were calculated to determine independent risk factors for MDRE UTI.

## 3. Results

### 3.1. Patient Characteristics

There were 676 patients with positive urine cultures identified during the study period; 184 who had asymptomatic bacteriuria or urinary tract colonization were excluded. Of the remaining 492 patients, 61 had UTIs due to non-Enterobacteriaceae bacteria (24 *Enterococcus *spp., 18 *Pseudomonas aeruginosa*, 15 *Staphylococcus aureus*, 3 *Staphylococcus saprophyticus*, and one *Stenotrophomonas maltophilia*) and were excluded. The study population consisted of 431 patients with Enterobacteriaceae UTIs. Demographics, comorbidities, types of UTIs, and history of antibiotic use are summarized in Table 1. The median age was 44 years (range 14–101 years) and the majority of patients were female (81%) and African American (52%). Obstructive uropathy was present in 59 (14%) patients and was secondary to urinary tract calculi, cervical cancer, bladder cancer, vaginal cancer, benign prostate hypertrophy, and ureteropelvic junction obstruction. Prior antibiotic use within the last 3 months was documented for 114 of 431 patients (27%) and fluoroquinolones were the most commonly used antibiotic (12%). Lower UTIs were diagnosed in 280 (65%) patients. Eighty-three patients (19%) had MDRE UTIs, while 348 patients (81%) had non-MDRE UTIs. Of these 431 patients with Enterobacteriaceae UTIs, 306 (71%) were treated as outpatients and 125 (29%) were hospitalized.

### 3.2. Prevalence and Antibiotic Resistance of Enterobacteriaceae

There were 451 non-duplicate Enterobacteriaceae isolates recovered from the 431 patients (some of these patients had UTIs caused by more than 1 organism in the same episode). The distribution of the bacteria in patients with and without MDRE UTIs is shown in Table 2. The majority of uropathogens were *Escherichia coli* [323 (72%)], *Klebsiella* spp. (66 (15%)), and *Proteus* spp. (32 (7%)). *Klebsiella* spp. was significantlymore common in patients with non-MDRE UTIs than those with MDRE UTIs (17% versus 5%; *P* = 0.002), while* Serratia* spp. and *Providencia* spp. were only identified in patients with MDRE UTIs. Rates of antibiotic resistance for Enterobacteriaceae are summarized in Table 3. The overall resistance rate for TMP-SMX, levofloxacin and nitrofurantoin was 24%, 17% and 14%, respectively. Antibiotic resistance rates for MDRE UTIs were significantly greater than non-MDRE UTIs for the majority of tested antibiotics including levofloxacin, 72% versus 14%; TMP-SMX, 77% versus 12%; gentamicin, 47% versus 1%; amoxicillin-clavulanate, 35% versus 4%; nitrofurantoin, 21% versus 12% and ceftriaxone, 20% versus 0%. All of the Enterobacteriaceae isolates were ertapenem susceptible (MIC ≤ 2 mg/L).

### 3.3. Resistance Pattern of MDRE

Among the 86 unique MDRE isolates, 21 (24%) isolates were ESBL producers. In regards to resistance pattern by antibiotic categories, the three most common patterns were combined PBI, fluoroquinolone and TMP-SMX resistance [18/86 (21%)], combined PBI, TMP-SMX and aminoglycoside resistance [11/86 (13%)] and combined PBI, fluoroquinolone, TMP-SMX and aminoglycoside resistance [8/86 (9%)].

### 3.4. Risk Factor for MDRE UTIs

Risk factors associated with MDRE UTIs were analyzed by comparing patients with and without MDRE UTIs. By univariate analysis [Table 1], age, male gender, diabetes mellitus, obstructive uropathy, healthcare-associated risks, prior UTI, and prior use of any antibiotics, penicillins, cephalosporins, fluoroquinolones, or TMP-SMX within 3 months were associated with MDRE UTIs. In the multivariate logistic regression analysis [Table 4], independent risk factors for MDRE were prior fluoroquinolone use within 3 months (aOR 3.64; 95% CI 1.74–7.64; *P* = 0.001], healthcare-associated risks (aOR 2.32; 95% CI 1.23–4.37; *P* = 0.009), and obstructive uropathy (aOR 2.22; 95% CI 1.04–4.78; *P* = 0.04). 

Based on in vitro antibiotic susceptibility testing, 61 of the 431 (13%) patients received empiric treatment with an antibiotic that tested nonsusceptible. Of these 61 patients, 31 (51%) received levofloxacin. Patients with MDRE UTIs were significantly more likely to receive a nonsusceptible antibiotic compared to those with non-MDRE UTIs (39% versus 8%; *P* less than 0.001).

## 4. Discussion

Our study found that the Enterobacteriaceae are the major causes of UTIs (88%) among ED patients and *E. coli *remains the most common pathogen in both MDRE and non-MDRE UTIs. We found a surprisingly high prevalence of MDRE UTIs among ED patients, 19% overall, and the prevalence of ESBL-associated UTIs was 4.6% of all UTIs which is comparable to the results of previous studies [4, 16]. A Chicago study of outpatient UTIs reported an increase in prevalence of ESBL-producing *E. coli *UTIs from 0.2% in 2003 to 3.0% in 2008 [4]. High rates of MDRE UTIs have also been reported in other US cities and overseas [16]. In the Americas, studies have focused mainly on the prevalence of ESBL-associated UTIs which are a subset of MDRE UTIs. The prevalence of ESBL community onset UTI has been as high as 30% in some Latin America countries [17].

Among non-MDRE UTIs, all tested antibiotics in this study, except ampicillin, were suitable for empiric treatment while the appropriate empiric therapy for MDRE UTIs was limited to carbapenems (100% susceptible) and amikacin (99% susceptible). In an outpatient setting like the ED, ertapenem is a particularly attractive option for treatment of MDRE UTIs due to its convenient once daily dosing. The drug can be administered intravenously or intramuscularly at other facilities near patients' residence or, if applicable, by trained family members. The efficacy of ertapenem 1 g daily was found to be comparable to once daily ceftriaxone in two well-designed studies that looked at the microbiologic eradication rates in patients with complicated urinary tract infections [18, 19]. The drug has a good safety profile and rarely causes hypersensitivity reactions or antibiotic-associated diarrhea. In addition, ertapenem is stable to hydrolysis by several types of beta-lactamases including ESBLs and concentrates well in the urine [20]. We found that ceftriaxone, another once daily antibiotic that is commonly used to treat UTIs, cannot be recommended for the treatment of MDRE UTIs given the high rate of resistance (20%). Amikacin demonstrated high level of in vitro susceptibility and could be used for a short course empiric therapy while awaiting the urine culture result. However, the risk of nephrotoxicity and ototoxicity, particularly in the elderly whose creatinine levels may underestimate the true glomerular filtration rate, should be borne in mind. In addition, clinicians are generally reluctant to use even a single dose of aminoglycoside in patients with active kidney disease or in patients receiving concomitant nephrotoxic drugs and prefer to reserve amikacin for the treatment of multidrug-resistant *Pseudomonas aeruginosa. *Unfortunately, there are few oral options for the treatment of MDRE UTIs and we found that oral antibiotic therapy is best guided by formal susceptibility results from urine cultures. Fosfomycin represents a promising option as it is active against ESBLs and is well tolerated with excellent urinary penetration [20], although this antibiotic was not assessed in our study. 

 One of the significant findings of our study is the importance of identifying patients with risk factors for MDRE UTIs in order to guide empiric drug selection. Common risk factors for MDRE-associated infections are not well-defined since most studies focus on particular subgroups of MDRE including ESBLs, KPC and NDM-1-associated infections or specific organism types, such as *E. coli *and *Klebsiella* spp. Previously-reported risk factors associated with infections caused by ESBL-producing Enterobacteriaceae include length of hospital and intensive care unit stay, presence of central venous or arterial catheters, emergency abdominal surgery, prior administration of antibiotic, especially second and third generation cephalosporins, prior residence in a long-term care facility, presence of a urinary catheter, ventilatory assistance, and chronic hemodialysis [21]. Poor functional status, ICU stay, and receipt of antibiotics, particularly fluoroquinolones, broad spectrum cephalosporins, and carbapenems were independently associated with KPC-associated infections [22], while risk factors for NDM-1-associated infections were travel and/or undergoing medical procedures in the Indian subcontinent [6]. We identified prior fluoroquinolone use within 3 months, healthcare-associated risks, including presence of chronic indwelling urinary catheters, healthcare exposure including hospital stay for at least 48 hours, nursing home or long-term care facility residence, regular hemodialysis clinic visits or recent urological procedures within the past 3 months, and obstructive uropathy as independent risk factors associated with MDRE UTIs. Most of these risk factors were consistent with previously reported ESBL-associated risks. However, we also identified hitherto unreported risk factors for MDRE UTIs including prior fluoroquinolone use, recent urological procedures, and obstructive uropathy. As recent fluoroquinolone use has been reported to be associated with quinolone-resistant UTIs [23], it is not surprising that we identified it as a risk factor for MDRE UTI given the well-recognized “collateral damage” associated with fluoroquinolone use [24]. Urological procedures and obstructive uropathy are risk factors for recurrent UTIs which likely result in multiple prior antibiotic exposures and could explain the increased risk of MDRE UTIs among patients with these characteristics.

In this study, we found that the majority of the MDRE isolates (76%) did not have an ESBL resistance pattern. Among the non-ESBL isolates, fluoroquinolone resistance was a common component of multidrug resistance. These findings correspond with the high rate of fluoroquinolone resistance reported in this study. Although fluoroquinolones are recommended for empiric therapy of complicated UTIs due to the previously reported low rates of resistance in the United States [1, 2, 25], our data suggests that fluoroquinolones are not appropriate empiric therapy in our ED for patients with risk factors for MDRE UTIs. In addition, our findings emphasize the importance of local antibiotic resistance patterns and risk factors assessment to guide appropriate therapy. 

Limitations of our study include the retrospective chart review nature of the study and the limited generalizability of our findings to other settings with different local susceptibility patterns and patient populations. In addition, the study was not designed to evaluate clinical and microbiological outcomes in patients receiving either active or inactive (based on in vitro susceptibility testing) antibiotic therapy. However, our findings suggest the need for research on uropathogen susceptibility pattern to direct appropriate therapy in each individual ED setting.

Regularly updated surveillance of local microbial prevalence and resistance patterns are needed to guide the empiric therapy for UTIs. In our ED setting, ertapenem may be appropriate therapy for outpatients with suspected or proven MDRE UTI. We suggest that all patients with risk factors for MDRE UTIs should have urine cultures performed to direct therapy.

## Figures and Tables

**Table 1:** Characteristics of patients with urinary tract infections caused by multidrug-resistant (MDR) and non-MDR Enterobacteriaceae.

Characteristics	All patients (*N* = 431)	MDR group (*N* = 83)	Non-MDR group (*N* = 348)	*P* ^a^
Median age (IQR) (years)	44 (27–68)	61 (33–82)	41 (26–62)	**<0.001**
Male gender	80 (19)	22 (26)	58 (17)	**0.04**
Ethnicity				
African American	226 (52)	38 (46)	188 (54)	0.18
Caucasian	96 (22)	20 (24)	76 (22)	0.66
Hispanic	93 (22)	22 (27)	71 (20)	0.22
Asian	6 (1)	2 (2)	4 (1)	0.33
Other	10 (2)	1 (1)	9 (3)	0.70
Pregnancy	19 (4)	1 (1)	18 (5)	0.14
Comorbidities				
Diabetes mellitus	87 (20)	25 (30)	62 (18)	**0.01**
Renal transplant	34 (8)	5 (6)	29 (8)	0.48
Obstructive uropathy	59 (14)	24 (29)	35 (10)	**<0.001**
Healthcare-associated risks^b^	143 (33)	51 (61)	92 (26)	**<0.001**
Prior urinary tract infection	172 (40)	53 (64)	119 (34)	**<0.001**
Prior antibiotic use within 3 months				
Any antibiotics	114 (27)	40 (48)	74 (21)	**<0.001**
Fluoroquinolones	50 (12)	27 (33)	23 (7)	**0.02**
Cephalosporins	38 (9)	13 (16)	25 (7)	**0.01**
Penicillins	31 (7)	11 (13)	20 (6)	**<0.001**
Vancomycin	26 (6)	8 (10)	18 (5)	0.11
TMP-SMX	18 (4)	8 (10)	10 (3)	**0.006**
Nitrofurantoin	14 (3)	5 (6)	9 (3)	0.09
Clindamycin	11 (3)	3 (4)	8 (2)	0.13
Carbapenems	6 (1)	3 (4)	3 (1)	0.25
Macrolides	5 (1)	2 (2)	3 (1)	0.45
Type of urinary tract infection				0.45
Lower tract disease	280 (65)	51 (61)	229 (66)	
Upper tract disease	151 (35)	32 (39)	119 (34)	

Data are in number (%) unless otherwise indicated.

^a^Compared between MDR and non-MDR groups.

^b^Defined as presence of chronic indwelling urinary catheters, healthcare exposure including hospital stay for at least 48 hours, nursing home or long-term care facility residence, regular hemodialysis clinic visits or urological procedures within the past 3 months.

IQR: interquartile range; TMP-SMX: trimethoprim-sulfamethoxazole.

**Table 2:** Distribution of 451 unique Enterobacteriaceae bacteria with and without multidrug resistance (MDR) isolated from 431 study patients.

Enterobacteriaceae bacteria	All isolates (*N* = 451)	MDR isolates (*N* = 86)	Non-MDR isolates (*N* = 365)	*P* ^a^
*Escherichia coli *	323 (72)	65 (76)	258 (71)	0.37
*Klebsiella* spp.	66 (15)	4 (5)	62 (17)	**0.002**
*Proteus mirabilis *	32 (7)	5 (6)	27 (7)	0.61
*Citrobacter* spp.	17 (4)	5 (6)	12 (3)	0.27
*Enterobacter* spp.	9 (2)	3 (3)	6 (2)	0.38
*Serratia* spp.	2 (0.4)	2 (2)	0 (0)	—
*Providencia* spp.	2 (0.4)	2 (2)	0 (0)	—

Data are in number (%) unless otherwise indicated.

^a^Compared between MDR and non-MDR isolates.

**Table 3:** Antibiotic resistance rates among 451 unique Enterobacteriaceae bacteria with and without multidrug resistance (MDR) isolated from 431 study patients.

Antibiotics	All isolates (*N* = 451)	MDR isolates (*N* = 86)	Non-MDR isolates (*N* = 365)	*P* ^a^
Ampicillin	55%	99%	45%	**<0.001**
Amoxicillin-clavulanate	10%	35%	4%	**<0.001**
Piperacillin-tazobactam	2%	9%	1%	**<0.001**
Cefuroxime	8%	38%	1%	**<0.001**
Ceftriaxone	4%	20%	0%	**<0.001**
Cefepime	3%	17%	0%	**<0.001**
Ertapenem	0%	0%	0%	1.00
Levofloxacin	17%	72%	4%	**<0.001**
Gentamicin	10%	47%	1%	**<0.001**
Amikacin	1%	2%	0.3%	0.10
Trimethoprim-sulfamethoxazole	24%	77%	12%	**<0.001**
Nitrofurantoin	14%	21%	12%	**0.04**
ESBL resistance pattern	5%	24%	0%	**<0.001**

^a^Compared between MDR and non-MDR isolates.

ESBL: extended-spectrum beta-lactamase.

**Table 4:** Risk factors associated with multidrug-resistant Enterobacteriaceae-associated urinary tract infections.

Risk factors	Univariate analysis		Multivariate analysis	
	OR (95% CI)	*P*	aOR (95% CI)	*P*
Age	1.02 (1.01–1.04)	**<0.001**	1.01 (1.00–1.02)	0.14
Male gender	1.80 (1.03–3.17)	**<0.001**	0.84 (0.41–1.69)	0.62
Diabetes mellitus	1.99 (1.16–3.42)	**0.01**	1.40 (0.75–2.63)	0.29
Obstructive uropathy	3.64 (2.02–6.56)	**<0.001**	2.22 (1.04–4.78)	**0.04**
Prior urinary tract infection	3.40 (2.06–5.60)	**<0.001**	1.73 (0.97–3.08)	0.06
Healthcare-associated risks^a^	4.44 (2.68–7.32)	**<0.001**	2.32 (1.23–4.37)	**0.009**
Prior penicillin use within 3 months	2.51 (1.15–5.46)	**0.02**	0.66 (0.25–1.71)	0.39
Prior cephalosporin use within 3 months	2.40 (1.17–4.92)	**0.01**	0.78 (0.32–1.89)	0.59
Prior fluoroquinolone use within 3 months	6.81 (3.65–12.72)	**<0.001**	3.64 (1.74–7.64)	**0.001**
Prior TMP-SMX use within 3 months	3.61 (1.38–9.44)	**0.006**	1.16 (0.35–3.84)	0.81

^a^Defined as presence of chronic indwelling urinary catheters, healthcare exposure including hospital stay for at least 48 hours, nursing home or long-term care facility residence, regular hemodialysis clinic visits or urological procedures within the past 3 months.

aOR: adjusted odds ratio; CI: confidence interval; OR: odds ratio; TMP-SMX: trimethoprim-sulfamethoxazole.

## References

[1] Gupta K, Hooton TM, Naber KG (2011). International clinical practice guidelines for the treatment of acute uncomplicated cystitis and pyelonephritis in women: a 2010 update by the infectious diseases society of America and the European society for microbiology and infectious diseases. *Clinical Infectious Diseases*.

[2] Hooton TM, Bradley SF, Cardenas DD (2010). Diagnosis, prevention, and treatment of catheter-aassociated urinary tract infection in adults: 2009 international clinical practice guidelines from the infectious diseases society of America. *Clinical Infectious Diseases*.

[3] Sanchez GV, Master RN, Karlowsky JA, Bordon JM (2012). In vitro antimicrobial resistance of urinary *Escherichia coli* isolates among U.S. outpatients from 2000 to 2010. *Antimicrobial Agents and Chemotherapy*.

[4] Qi C, Pilla V, Yu JH, Reed K (2010). Changing prevalence of *Escherichia coli* with CTX-M-type extended-spectrum *β*-lactamases in outpatient urinary E. coli between 2003 and 2008. *Diagnostic Microbiology and Infectious Disease*.

[5] Gupta N, Limbago BM, Patel JB, Kallen AJ (2011). Carbapenem-resistant enterobacteriaceae: epidemiology and prevention. *Clinical Infectious Diseases*.

[6] Kumarasamy KK, Toleman MA, Walsh TR (2010). Emergence of a new antibiotic resistance mechanism in India, Pakistan, and the UK: a molecular, biological, and epidemiological study. *The Lancet Infectious Diseases*.

[7] Paterson DL, Ko W-C, Von Gottberg A (2004). Antibiotic therapy for *Klebsiella pneumoniae* bacteremia: implications of production of extended-spectrum *β*-lactamases. *Clinical Infectious Diseases*.

[8] Abrahamian FM, Moran GJ, Talan DA (2008). Urinary tract infections in the emergency department. *Infectious Disease Clinics of North America*.

[9] Horan TC, Andrus M, Dudeck MA (2008). CDC/NHSN surveillance definition of health care-associated infection and criteria for specific types of infections in the acute care setting. *The American Journal of Infection Control*.

[10] 10. American Thoracic Society and Infectious Diseases Society of America (2005). Guidelines for the management of adults with hospital-acquired, ventilator-associated, and healthcare-associated pneumonia. *American Journal of Respiratory and Critical Care Medicine*.

[11] Clinical and Laboratory Standards Institute Performance standards for antimicrobial susceptibility testing. Nineteenth informational supplement. Approved standard M100-S19. *Clinical and Laboratory Standards Institute*.

[12] Cohen-Nahum K, Saidel-Odes L, Riesenberg K, Schlaeffer F, Borer A (2010). Urinary tract infections caused by multi-drug resistant proteus mirabilis: risk factors and clinical outcomes. *Infection*.

[13] Magiorakos A-P, Srinivasan A, Carey RB (2012). Multidrug-resistant, extensively drug-resistant and pandrug-resistant bacteria: an international expert proposal for interim standard definitions for acquired resistance. *Clinical Microbiology and Infection*.

[14] Jacoby GA (1998). Epidemiology of extended-spectrum beta-lactamases. *Clinical Infectious Diseases*.

[15] Gudiol C, Tubau F, Calatayud L (2011). Bacteraemia due to multidrug-resistant gram-negative bacilli in cancer patients: risk factors, antibiotic therapy and outcomes. *Journal of Antimicrobial Chemotherapy*.

[16] Diekema DJ, BootsMiller BJ, Vaughn TE (2004). Antimicrobial resistance trends and outbreak frequency in United States hospitals. *Clinical Infectious Diseases*.

[17] Bours PHA, Polak R, Hoepelman AIM, Delgado E, Jarquin A, Matute AJ (2010). Increasing resistance in community-acquired urinary tract infections in Latin America, five years after the implementation of national therapeutic guidelines. *International Journal of Infectious Diseases*.

[18] Jimenez-Cruz F, Jasovich A, Cajigas J (2002). A prospective, multicenter, randomized, double-blind study comparing ertapenem and ceftriaxone followed by appropriate oral therapy for complicated urinary tract infections in adults. *Urology*.

[19] Tomera KM, Burdmann EA, Pamo Reyna OG (2002). Ertapenem versus ceftriaxone followed by appropriate oral therapy for treatment of complicated urinary tract infections in adults: results of a prospective, randomized, double-blind multicenter study. *Antimicrobial Agents and Chemotherapy*.

[20] Prakash V, Lewis JS, Herrera ML, Wickes BL, Jorgensen JH (2009). Oral and parenteral therapeutic options for outpatient urinary infections caused by enterobacteriaceae producing ctx-m extended-spectrum *β*-lactamases. *Antimicrobial Agents and Chemotherapy*.

[21] Jacoby GA, Munoz-Price LS (2005). The new beta-lactamases. *The New England Journal of Medicine*.

[22] Schwaber MJ, Klarfeld-Lidji S, Navon-Venezia S, Schwartz D, Leavitt A, Carmeli Y (2008). Predictors of carbapenem-resistant *Klebsiella pneumoniae* acquisition anions hospitalized adults and effect of acquisition on mortality. *Antimicrobial Agents and Chemotherapy*.

[23] Johnson L, Sabel A, Burman WJ (2008). Emergence of fluoroquinolone resistance in outpatient urinary *Escherichia coli* isolates. *The American Journal of Medicine*.

[24] Paterson DL (2004). “Collateral damage” from cephalosporin or quinolone antibiotic therapy. *Clinical Infectious Diseases*.

[25] Zhanel GG, Hisanaga TL, Laing NM (2005). Antibiotic resistance in outpatient urinary isolates: final results from the North American Urinary Tract Infection Collaborative Alliance (NAUTICA). *International Journal of Antimicrobial Agents*.

